# *Leishmania major-*derived lipophosphoglycan influences the host’s early immune response by inducing platelet activation and DKK1 production via TLR1/2

**DOI:** 10.3389/fimmu.2023.1257046

**Published:** 2023-10-11

**Authors:** Olivia C. Ihedioha, Anutr Sivakoses, Stephen M. Beverley, Diane McMahon-Pratt, Alfred L. M. Bothwell

**Affiliations:** ^1^ 1Department of Immunobiology, College of Medicine, University of Arizona, Tucson, AZ, United States; ^2^ Department of Molecular Microbiology, Washington University School of Medicine in St Louis, St. Louis, MI, United States; ^3^ Department of Epidemiology of Microbial Diseases, Yale School of Public Health, New Haven, CT, United States

**Keywords:** Leishmaniasis, P-selectin, innate response, leukocyte-platelet aggregates, platelet

## Abstract

**Background:**

Platelets are rapidly deployed to infection sites and respond to pathogenic molecules via pattern recognition receptors (TLR, NLRP). Dickkopf1 (DKK1) is a quintessential Wnt antagonist produced by a variety of cell types including platelets, endothelial cells, and is known to modulate pro-inflammatory responses in infectious diseases and cancer. Moreover, DKK1 is critical for forming leukocyte-platelet aggregates and induction of type 2 cell-mediated immune responses. Our previous publication showed activated platelets release DKK1 following *Leishmania major* recognition.

**Results:**

Here we probed the role of the key surface virulence glycoconjugate lipophosphoglycan (LPG), on DKK1 production using null mutants deficient in LPG synthesis (*Δlpg1-* and *Δlpg2-*). *Leishmania*-induced DKK1 production was reduced to control levels in the absence of LPG in both mutants and was restored upon re-expression of the cognate *LPG1* or *LPG2* genes. Furthermore, the formation of leukocyte-platelet aggregates was dependent on LPG. LPG mediated platelet activation and DKK1 production occurs through TLR1/2.

**Conclusion:**

Thus, LPG is a key virulence factor that induces DKK1 production from activated platelets, and the circulating DKK1 promotes Th2 cell polarization. This suggests that LPG-activated platelets can drive innate and adaptive immune responses to *Leishmania* infection.

## Introduction

Cutaneous leishmaniasis (CL) is a zoonotic protozoan disease caused by over 15 different parasite species of *Leishmania* (1). Current data suggest that more than 1 billion people are at risk of cutaneous leishmaniasis, and more than 1 million new cases occur each year in approximately 90 countries worldwide (2). Cutaneous leishmaniasis is endemic in many regions of the world, including the Mediterranean, North Africa, Central America, the Middle East, and northern parts of South America (3). Most of the detected cases of leishmaniasis in the United States are amongst travellers to endemic regions (4). Therapeutic treatment for managing cutaneous leishmaniasis involves the use of a limited number of drugs, known to cause serious side effects (5). Thus, therapeutic approaches that promote healing and reduce drug toxicity are highly desired. Combinatorial therapy involving a combination of immunomodulatory molecules with current therapies has the potential of limiting drug toxicity (6). Therefore, there is a need to identify immunomodulatory mechanisms and compounds that will serve as a therapeutic target for controlling leishmaniasis. Identifying immunomodulatory molecules for controlling leishmaniasis requires understanding *Leishmania* surface component interaction with host immune cells.

Platelets are known to play a role in hemostasis and for their essential contribution to protection against infectious pathogens (7). By interaction with macrophage cells, monocytes, neutrophils, lymphocytes, and the endothelium, platelets are therefore important executors during inflammatory and immune responses (8, 9). The membrane glycoprotein CD62P, a member of the selectin family, is expressed on activated platelets (10) and is redistributed from the secretory α-granules (11). PSGL-1, one of the best-characterized selectin ligands, is mainly expressed in leukocytes (12). Leukocyte platelet aggregation formed by adhesion between activated platelet and mature leukocyte is mediated by CD62P and PSGL-1 (13–15). The formation of leukocyte platelet aggregation (LPA) is necessary for effective migration to the infection site (16), which might be induced by *L. major* surface membrane components.

Surface membrane components of pathogens (virus, bacteria, parasite) are important in initiation of infection and interaction with the host. The *Leishmania* parasite has a surface coat (glycocalyx) that is composed of glycosylated proteins and lipids that are crucial for the parasite’s survival, replication and pathogenesis in the insect vector and mammalian hosts (17, 18). These glycoconjugates on the parasite coat are differentially expressed in the different developmental stages of the *Leishmania* parasite (18). Of these molecules, lipophosphoglycan (LPG) and proteophosphoglycan (PPG) show structural similarities through the presence of the canonical phosphoglycan repeating units (Gal-Man-PO4) (19, 20). Thus, there is a strong potential for cross-activity of these shared motifs in biochemical or cell biological tests of purified molecules. This limitation can be overcome using *Leishmania major* rendered genetically deficient in either or both LPG or PPG. Parasites specifically lacking *LPG1* were obtained through homologous gene replacement, deleting the *LPG1* galactofuranosyl transferase required for synthesis of a critical linkage within the LPG glycan core domain (21). These mutants otherwise express normal levels of other surface virulence glycoconjugates including GPI anchored proteins, PPG and glycoinositolphospholipids (GIPLS) (22). Similarly, homologous gene replacement of LPG2 encoding the Golgi GDP-mannose nucleotide sugar transporter completely ablate PG repeat synthesis, rendering parasites deficient in both intact LPG and PPG (23). Extensive studies using these mutants have shown that LPG plays critical roles in pathogenesis and parasite survival, through the evasion of complement-mediate lysis, preventing natural killer T cells from recognizing the *Leishmania*-infected macrophage, suppression of oxidative burst response, macrophage-mediated killing and modulation of host immune responses (24–26). Further, LPG has the ability to alter dendritic cell function, which affects the host’s immunological responses by blocking antigen presentation and promoting an early IL-4 response (27).

We demonstrated activated platelets release of DKK1 following recognition of *L. major* (28). In that report, we found that DKK1 is maintained at a high concentration in *L. major* infected mice, and that depletion of platelets resulted in the complete loss of the DKK1 response to infection (28). Further inhibition of DKK1 activity resulted in a significant reduction in cells recruited to the site of infection. The DKK1 response was shown to be critical to the development of the Th2 response to infection through the induction of MAPK and mTOR signaling pathways. It has been reported that platelet-derived DKK1 is elevated in different diseases following the recognition of pathogen-associated molecular patterns through receptors other than TLR activation. However, no one so far has related DKK1 elevation to platelet-TLR activation or has identified leishmanial-associated molecules involved in DKK1 production.

In the current study, we expand upon our initial observations to demonstrate that *Leishmania major*-mediated platelet activation is initiated via the TLR1/2 signalling pathway, by the specific engagement of the leishmanial virulence factor, lipophosphoglycan (LPG). Overall, our studies demonstrate that LPG plays a critical role in Th2 induction, polymorphonuclear neutrophil (PMN) recruitment and the establishment of infection, through engagement and activation of platelets.

## Experimental procedures

### Mice

BALB/c mice (6 weeks old) were purchased from the Jackson Laboratory. All mice were housed at the University of Arizona Animal Care Facilities. All mouse protocols were approved by the Arizona University Institutional Animal Care and Use Committee (IACUC) in accordance with the Association for Assessment and Accreditation of Laboratory Animal Care International (AAALAC).

### Parasite strains and infection protocol

The *L. major* mutants (*Δlpg1*- and *Δlpg2*-) and add-backs (Δ*lpg1*-/+*LPG1* and Δ*lpg2*-/+*LPG2)* were derivatives of WT *L. major* LV39 clone 5 background. They were made by homologous gene targeting and maintained in selective media as described previously (22, 23, 29, 30). *Leishmania major* parasites were maintained at 26°C in M199 culture medium (Thermo Fisher Scientific) supplemented with 20% heat-inactivated FBS (Thermo Fisher Scientific)), 20 mM HEPES (Sigma-Aldrich) and 50 ug/ml gentamycin (Thermo Fisher Scientific). Prior to using parasites for infection, metacyclic promastigotes were isolated from stationary-phase cultures using density gradient centrifugation (31, 32). Metacyclic promastigotes were washed three times in cold phosphate-buffered saline (PBS) (Thermo Fisher Scientific) by centrifugation, resuspended in PBS at 2X10^8^/ml and 10 μl containing 2x10^6^ metacyclic promastigotes were injected into the top of the right hind footpad.

### Measurement of lesion size and estimation of parasite burden

Lesion size was measured weekly with Vernier calipers and determined by subtracting the size of the uninfected from that of the infected footpad. Parasite burden in the infected footpad was estimated by limiting dilution analysis as previously described (33).

### Platelet preparation and P-selectin expression

Blood was drawn by retro-orbital bleeding under isoflurane anesthesia (MWI Animal Health) into tubes containing 3.2% citrate buffer (G-Biosciences) from naïve mice or at days 3, 14 and 42 PI. Platelets were prepared as previously described (34). In brief, blood was diluted with 250 μl of modified Tyrode’s-HEPES buffer (134 mM NaCl, 0.34 mM Na_2_HPO_4_, 2.9 mM KCl, 12 mM NaHCO_3_, 20 mM HEPES, 5 mM glucose, and 1 mM MgCl_2_, pH 7.3) and centrifuged at 250 x *g* for 15 minutes at room temperature. Platelet-rich plasma was removed, and platelets were then isolated by centrifugation at 900 × *g* for 30 min. Plasma was collected for DKK1 ELISA.

*In vitro* stimulation of platelets was done using various stock concentrations of soluble leishmania antigen (SLAG) (1:25, 1:50, 1:200, 1:400), Pam2CSK4, Pam3CSK4 (InvivoGen), LPS (*Escherichia coli* O111:B4-Sigma) and control isotype antibodies (IgG1, IgG2a and IgG2b) (Invitrogen). DKK1 inhibition was done using anti-TLR4, anti-TLR2, anti-TLR1/2, anti-TLR2/6, anti-TLR1/2/6 antibody (Invitrogen), and Go 6976 PKC-alpha inhibitor (Abcam) before incubation for 1 hr at room temperature. Platelets were isolated by centrifugation at 550 x *g* for 10 minutes, and the supernatant was collected for DKK1 ELISA. For SLAG preparation, *L. major* parasites were prepared at 5 × 10^8^ parasites/ml in M199 medium (without FCS). SLAG was prepared by repeated five freeze and thaw cycles at -80°C and room temperature, respectively. SLAG supernatant was collected after centrifugation at 1500rpm for 20 minutes and stored at -80°C. For detection of LPG expression, Western Blot of SLAG (7 µg) was resolved by SDS-PAGE electrophoresis using 10% PAGE; molecules were transferred to PVDF membrane using the iBlot 2 semi-dry blotting system (Thermofisher Scientific Inc.). The membrane was blocked with 5% powdered milk in Tris Buffered Saline (TBS) and incubated for 1 hour at room temperature. Blots were probed with WIC 79.3 antibody (1:1000). After washing in TBS supplemented with 0.05% Tween 20 (TBST), the membrane was incubated for 1 h with anti-mouse IgG conjugated with Horseradish peroxidase (1:7500-Invitrogen) and the reaction was visualized using Pico PLUS Chemiluminescent substrate kit (Thermo Fisher Scientific). Signals were acquired using ChemiDoc Imaging System (Bio-Rad).

For FACS analyses, isolated platelets (10x10^6^/ml) were washed at 550 x *g* for 10 minutes in the presence of PGE_1_ (140 nM; Sigma-Aldrich) and indomethacin (10 μM; Thermo Fisher Scientific). Platelets were resuspended to the required density in modified Tyrode’s-HEPES buffer (Sigma-Aldrich) and rested for 30 minutes at 37°C in the presence of 10 μM indomethacin prior to staining. Staining for P-selectin expression was done using PE-conjugated CD41 (BioLegend) (to determine platelet purity) and APC-conjugated CD62P (Invitrogen; a marker of P- selectin). Stained platelets (1 × 10^6^/ml in Tyrode’s-HEPES buffer; 100 μl) were analyzed using a BD FACSCanto flow cytometer; analysis was done using FlowJo software. A total of 10,000 events per sample were collected. The mean fluorescent intensity of P-selectin was compared among the different groups in the overlay.

### Leukocyte-platelet aggregation

Leukocyte-platelet aggregation assessment was performed as described previously with minor modifications (28). Briefly, 100 μl blood was collected via the retro-orbital sinus into tubes containing 3.2% citrate buffer. Peripheral blood (10 µl) was stained with PE-conjugated anti-mouse CD41 antibody and Pacific blue conjugated CD45 antibody (BioLegend) for 10 mins in the dark at room temperature. Fix/red blood cell lysis solution (eBiosciences) was added and incubated for 15 min in the dark at room temperature. Samples were analyzed by flow cytometry within 4 to 6 hours. Live gating was performed on leukocyte-sized events to exclude single platelets. Leukocytes were identified by their forward and side scatter characteristics and CD45 expression. The CD41+ subpopulation identified leukocyte-platelet aggregates.

### DKK1 ELISA

Enzyme-linked immunosorbent assays were performed using a mouse DKK1 ELISA kit (Thermo Fisher Scientific) to determine the concentration of DKK1 in plasma and cell culture supernatants according to the manufacturer’s protocol.

### Statistical analyses

The *in vivo* expression of P-selectin and DKK1 production was analyzed using one-way ANOVA with Bonferroni’s *post hoc* test. Also, *in vitro* DKK1 production and P-selectin expression following SLAG stimulation was analyzed using one-way ANOVA with Bonferroni’s *post hoc* test. Comparison of neutralizing antibody inhibition of platelet DKK1 induced by SLAG obtained from WT, addback and mutant parasites was done statistically using Student’s t-test. Likewise, a comparison of PKC-alpha inhibition of DKK1 induced by SLAG obtained from WT, addback and mutant parasites was done statistically using one-way ANOVA with Bonferroni’s *post hoc* test. In addition, lesion size, parasitic load and percentage of LPA was analyzed using one-way ANOVA with Bonferroni’s *post hoc* test. Data presented as means ± standard errors were performed using GraphPad Prism software (GraphPad Software, San Diego, CA, USA).

## Results

### *Δlpg1*- and *Δlpg2*- parasites induce minimal expression of P-selectin in platelets

**Figure 1:**
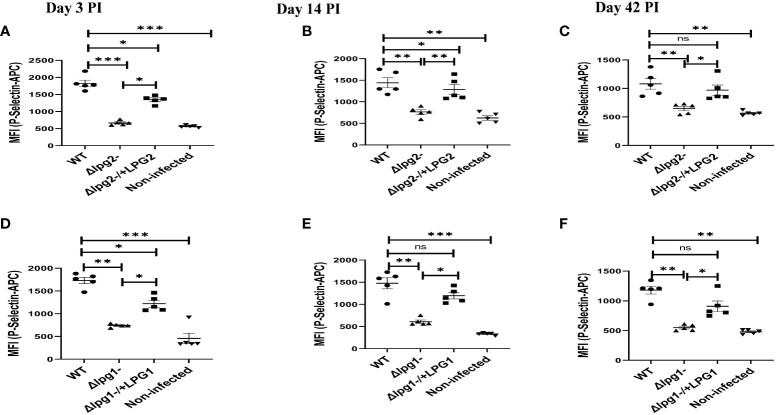
*Δlpg1*- and *Δlpg2*- induce minimal P-selectin expression in activated platelets. BALB/c mice were challenged with infective metacyclic promastigote (2 x 10^6^ parasites, n = 5) of WT, *Δlpg1-, Δlpg2*-, *Δlpg1*-/+*LPG1* and Δ*lpg2*-/+*LPG2* strains via the footpad. Control mice (n = 5) were given 0.9% NaCl saline. Blood was collected via retro-orbital sinus at days 3, 14 and 42 PI. Isolated platelet samples were analyzed by flow cytometry for P-selectin expression. In all the experiments, WT-infected and non-infected mice served as positive and negative controls, respectively. Each dot indicates the expression of P-selectin by CD41+ cells **(A–F)**. Results are presented as mean (± SEM) and are representative of 3 independent experiments. One-way ANOVA with Bonferroni’s *post hoc* test was performed to analyze the data *p < 0.05, **p < 0.01, ***p < 0.001, ‘ns’ indicates not significant (p > 0.05).

Platelets are rapidly deployed to sites of infection where they can modulate immune/inflammatory processes by secreting cytokines, chemokines, and other inflammatory mediators (35). P-selectin is an adhesion receptor for leukocytes expressed by activated platelets (36). To investigate the possible effects of *LPG1- and or LPG2-*dependent molecules on platelet activation *in vivo*, expression of P-selectin was determined from platelets obtained from mice that had been infected by WT, *Δlpg1*-, *Δlpg2^-^*, Δ*lpg1*-/+*LPG1* and Δ*lpg2* ^-^/+*LPG2* parasites for varying periods of time. Relative to the WT controls, P-selectin expression was significantly suppressed in platelets obtained from *Δlpg1*- and *Δlpg2*- mutant infected mice on days 3, 14 and 42 PI. As expected, mice infected with “add-back’ control parasites (Δ*lpg1*-/+*LPG1* and Δ*lpg2*-/+*LPG2)* manifested restoration of P-selectin expression similar to those infected with WT parasites (**Figures 1A–F**).

**Figure 2:**
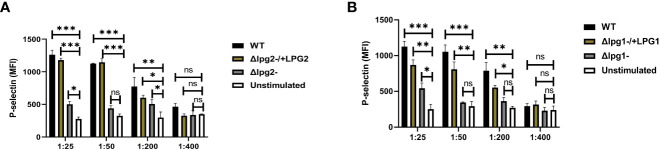
Less efficient expression of P-selectin from *Δlpg1*- and *Δlpg2*- SLAG-activated platelets. Platelets isolated from naïve mice were incubated with various concentrations of SLAG (derived from WT, *Δlpg1*-, Δ*lpg2*-, Δ*lpg1*-/+*LPG1* and Δ*lpg2*-/+*LPG2* strains) for 1hr. Platelet samples were analyzed by Flow cytometry for P-selectin expression. In all the experiments, WT-SLAG activated and non-activated platelets served as a positive and negative control, respectively. Column graphs **(A, B)** indicate the expression of P-selectin by CD41^+^ cells. Results are presented as mean +/- SEM of replicate wells and represent 3 independent experiments. Data are presented by comparing DKK1 production from the non-SLAG and SLAG (derived from WT, *Δlpg1*-, Δ*lpg2*-, Δ*lpg1*-/+*LPG1* and Δ*lpg2*-/+*LPG2* strains) activated platelets. One-way ANOVA with Bonferroni’s *post hoc* test was performed to analyze the data *p < 0.05, **p < 0.01, ***p < 0.001, ‘ns’ indicates not significant (p > 0.05).

We also characterized P-selectin expression *in vitro* using SLAG-stimulated platelets obtained from naïve mice. Stimulation of platelets obtained from naive mice with SLAG derived from *Δlpg1*- and *Δlpg2*- parasites induced poor P-selectin expression. In contrast, platelets stimulated with SLAG derived from WT, Δ*lpg1*-/+*LPG1* and Δ*lpg2*-/+*LPG2* parasites induced robust P-selectin expression (**Figures 2A, B**). Taken together, these data suggest that the inability of *Δlpg1*- and *Δlpg2*- parasites to induce P-selectin expression arises from a lack of *LPG1 or LPG2* dependent products. As *Δlpg2*- parasites lack all phosphoglycan-containing glycans (LPG, PPG and others) while *Δlpg1*- lacks only LPG, the lack of a significant difference between *Δlpg1*- and *Δlpg2*- mutants indicates that platelet activation and P-selectin expression are primarily regulated by LPG.

### *Δlpg1*- and *Δlpg2*- parasites are less efficient in inducing DKK1 production in platelets

**Figure 3:**
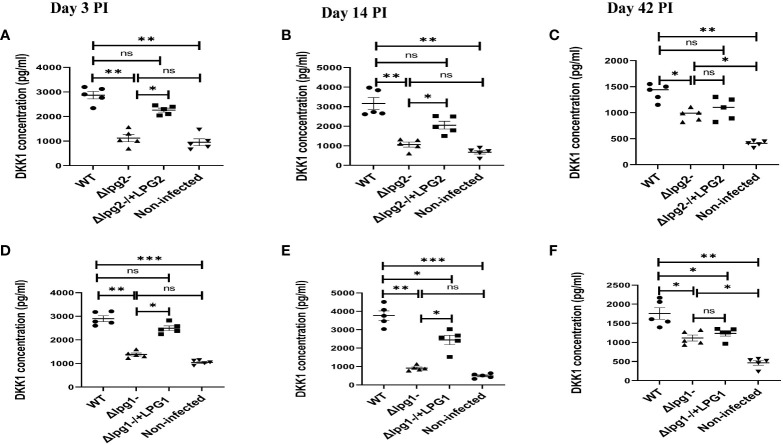
*Δlpg1*- and *Δlpg2*- parasites induce less plasma DKK1 production from the platelets. Six-week-old female BALB/c mice were challenged with infective metacyclic promastigote (2 x 10^6^ parasites, n = 5) of WT, *Δlpg1*-, Δ*lpg2*-, Δ*lpg1*-/+*LPG1* and Δ*lpg2*-/+*LPG2* strains via the footpad. Control mice (n = 5) were given 0.9% NaCl Saline. Blood was collected via retro-orbital sinus at days 3, 14 and 42 PI. Plasma samples were analyzed by ELISA For DKK1 production **(A–F)**. In all experiments, WT-infected and non-infected mice served as positive and negative controls, respectively. Results are presented as mean +/- SEM of replicate wells and are representative of 3 independent experiments. One-way ANOVA with Bonferroni’s *post hoc* test was performed to analyze the data *p < 0.05, **p < 0.01, ***p < 0.001, ‘ns’ indicates not significant (p > 0.05).

We previously showed that activated platelets release DKK1 following recognition of *L. major* (28). Furthermore, DKK1 is maintained at a high concentration in *L. major* infected mice; depletion of platelets resulted in the complete loss of DKK1 (28). To identify the *L. major* ligand involved in DKK1 production, we first compared the effect of *LPG1* and *LPG2* gene-dependent molecules in plasma DKK1 production at days 3, 14 and 42 PI. We confirmed a significantly decreased production of plasma DKK1 in *Δlpg1*- and *Δlpg2*- mutant infected mice compared to WT controls, but in Δ*lpg1*-/+*LPG1* and Δ*lpg2*-/+*LPG2* infected mice, DKK1 production was restored (**Figures 3A–F**). Compared to days 3 and 14 PI, there was a reduction in the concentration of DKK1 produced by activated platelets on day 42 PI.

**Figure 4:**
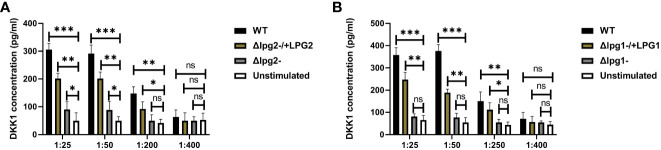
Poor induction of DKK1 from *Δlpg1*- and *Δlpg2*- SLAG-activated platelets. Platelets from naïve mice were incubated with various concentrations of SLAG (derived from WT, *Δlpg1*-, Δ*lpg2*-, Δ*lpg1*-/+*LPG1* and Δ*lpg2*-/+*LPG2* strains) for 1hr. Cell culture supernatant samples were analyzed by ELISA for DKK1 production as shown in the column graphs **(A, B)**. In all experiments, WT-SLAG activated, and non-activated platelets served as positive and negative controls, respectively. Results are presented as mean +/- SEM of replicate wells and represent 3 independent experiments. Data are presented by comparing the DKK1 production from non-SLAG and SLAG (derived from WT, *Δlpg1*-, Δ*lpg2*-, Δ*lpg1*-/+*LPG1* and Δ*lpg2*-/+*LPG2* strains) activated platelets. One-way ANOVA with Bonferroni’s *post hoc* test was performed to analyze the data *p < 0.05, **p < 0.01, ***p < 0.001, ‘ns’ indicates not significant (p > 0.05).

Impaired DKK1 production from the *Δlpg1^-^* and *Δlpg2*- were further confirmed via an *in vitro* stimulation of platelets with SLAG obtained from *Δlpg1*- and *Δlpg2*- parasites. Consistent with the plasma DKK1, *in vitro* stimulation of naïve platelets with SLAG obtained from *Δlpg1*- and *Δlpg2*- parasites failed to induce significant production of DKK1, in contrast a significant level of DKK1 was released by platelets stimulated with SLAG derived from WT *L. major* (**Figures 4A, B**) or the genetically reconstituted organisms. Since the levels of DKK1 produced in *Δlpg1*- and *Δlpg2*- infected mice are comparable, these data suggest that activated platelets release DKK1, a process which depends on the leishmania-derived LPG virulence factor; PPG does not appear to additionally contribute to platelet activation.

### Significant reduction in lesion size and parasitic burden observed in *Δlpg1*- and *Δlpg2*- infected mice was restored in mice infected with Δ*lpg1*-/+*LPG1* and Δ*lpg2*-/+*LPG2* parasites

**Figure 5:**
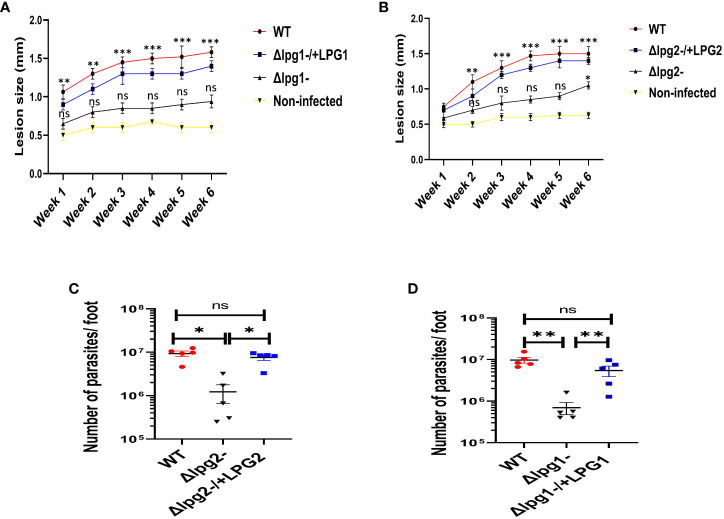
*Δlpg1*- and *Δlpg2*- parasites differ from specific add-backs and WT parasites in their capacity to induce lesions and increase the parasite burdens. The infected foot from each mouse in WT, *Δlpg1*-, Δ*lpg2*-, Δ*lpg1*-/+*LPG1* and Δ*lpg2*-/+*LPG2* parasite-infected groups were measured for lesion size weekly using a vernier caliper **(A, B)**, and parasite burden (at day 42 PI) was determined by limiting dilution assay **(C, D)**. Results are presented as mean +/- SEM. For Figures **(A, B)**, mice in all the infected groups were compared with the non-infected group and data analysis was done using one-way ANOVA with Bonferroni’s *post hoc* test *p < 0.05, **p < 0.01, ***p < 0.001, ‘ns’ indicates not significant (p > 0.05).

The *Δlpg1*- and *Δlpg2*- parasites had a greatly delayed formation of lesions compared with WT controls, whereas lesions produced by the Δ*lpg1*-/+*LPG1* and Δ*lpg2*-/+*LPG2* parasites showed minimal delay and resembled WT controls (**Figures 5A, B**). In addition, the parasitic burden was significantly decreased in *Δlpg1*- and *Δlpg2*- infected mice compared to WT controls, and restoration of the *LPG1* and *LPG2* genes resulted in parasite survival, which is comparable with the WT controls (**Figures 5C, D**). The pattern of lesion size in the footpads and parasitic burdens of mice infected with genetically deficient *Leishmania* (*LPG1* or *LPG2* gene) were comparable (**Figures 5A–D**). These data are consistent with previous publications concerning the virulence of these *lpg* mutant parasites (25, 29, 37). Repetition of this experiment serves as a confirmation of these results for the mutant lines used in the study.

### Decrease production of DKK1 in SLAG-stimulated platelets in the presence of PKC-alpha inhibitor and anti-TLR1/2 antibody

**Figure 6:**
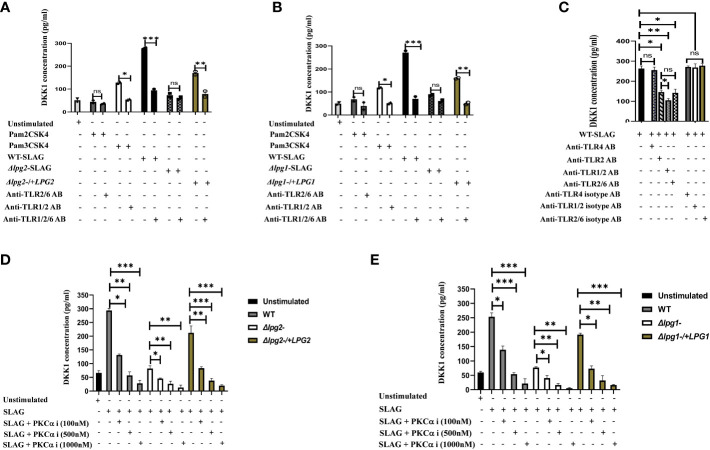
Anti-TLR1/2 antibody and PKC-alpha inhibitor significantly decreased DKK1 production in SLAG-activated platelets. Inhibition of DKK1 by neutralizing antibodies (10 μg/ml) following 1hr incubation with SLAG (derived from WT, *Δlpg1*-, Δ*lpg2*-, Δ*lpg1*-/+*LPG1* and Δ*lpg2*-/+*LPG2* strains) activated platelets. SLAG concentration is 1:50. In all experiments, Pam2CSK4 (10 μg/ml), Pam3CSK4 (10 μg/ml) and unstimulated samples served as a positive and negative control, respectively **(A, B)**. Inhibition of DKK1 by neutralizing antibodies (10 μg/ml) following 1hr incubation with WT SLAG (1:50) activated platelets **(C)**. Inhibition of DKK1 by PKC-alpha inhibitor (100nM, 500nM and 1000nM) following 1hr incubation with SLAG (derived from WT, *Δlpg1*-, Δ*lpg2*-, Δ*lpg1*-/+*LPG1* and Δ*lpg2*-/+*LPG2* strains) activated platelets **(D, E)**. Results are presented as mean +/- SEM of replicate wells and represent 3 independent experiments. For Figures **(A, B)**, Student’s t-test was performed, while one-way ANOVA with Bonferroni’s *post hoc* test was performed to analyze the data in Figures **(C–E)**. *p < 0.05, **p < 0.01, ***p < 0.001, ‘ns’ indicates not significant (p > 0.05). Note that the DKK1 production from Δlpg1- and Δlpg2- SLAG-stimulated platelets was found to be background platelet DKK1, as non-stimulated platelets showed comparable levels of DKK1 release/PKC-alpha inhibition (**Figure S2**).

Platelets express functional TLR2/4 and MyD88, which participate in platelet responsiveness to infection (38). We previously showed that activated platelets release DKK1 following recognition of *L. major* and PKC-alpha inhibitor or neutralization of TLR2 blocks secretion of DKK1 from SLAG-stimulated human platelets (28). PKC-alpha inhibitor is known to block the TLR2/MyD88 signalling pathway (39). An important feature of TLR2 is the ability to form heterodimers with its co-receptors (TLR1 and TLR6). Therefore, to confirm that TLR1/2/6 recognizes *Leishmania*-derived LPG and initiates the production of DKK1, neutralizing antibodies were used to inhibit the function of these specific TLRs in the presence of SLAG. Exposure of *Δlpg1*- and *Δlpg2*- SLAG-stimulated platelets to blocking anti-TLR1/2/6 antibodies showed no significant effect, but significant inhibition of DKK1 production by anti TLR1/2/6 antibodies was observed in WT, Δ*lpg1*-/+*LPG1* and Δ*lpg2*-/+*LPG*2 SLAG treated platelets (**Figures 6A, B**). To determine the level of DKK1 inhibition by the neutralizing antibodies, platelets were treated with neutralizing antibodies (anti-TLR2, TLR1/2 and TLR2/6) or control isotype antibodies (IgG2a and IgG1) in the presence of WT-derived SLAG. Results showed that neutralization of TLR1/2/6 with an anti-TLR1/2/6 mAb markedly reduced SLAG-induced DKK1 production in platelets compared with control isotype antibodies. Relative to DKK1 inhibition by anti-TLR2 blocking antibody, addition of anti-TLR1 (TLR1/2) antibody significantly deceased DKK1 production, while addition of anti-TLR6 antibody had no significant effect (**Figure 6C**). This suggests that only TLR1/2 played a primary role in LPG induced DKK1 production. Previous studies reported that inhibition of TLR2 and 4 attenuates inflammatory response and parasite burden in cutaneous leishmaniasis (40). Therefore, to determine whether TLR4 contributes in DKK1 production after LPG recognition by platelets, platelets were treated with anti-TLR4 neutralizing antibody or control isotype antibody (IgG2b) in the presence of WT-derived SLAG. Results showed that neutralization of TLR4, with an anti-TLR4 mAb failed to reduce SLAG-induced DKK1 production. Also, there was no change in DKK1 production in platelets treated with the control isotype antibody. This suggests that TLR4 played no role in LPG induced DKK1 production (**Figure 6C**). To confirm the specificity of this data, we assessed the possibility of other PAMPs (not directly related to LPG) to induce DKK1 release. Platelets treated with LPS (TLR4) and Pam2CSK4 (TLR2/6) failed to elicit DKK1 production. However, Pam3CSK4 (TLR1/2) slightly induced DKK1. This suggests that LPS and Pam2CSK4 lack the ability to induce DKK1 production (**Figure S3**). To verify that SLAG preparations of the mutant lines lack LPG, Western blot assay of SLAG preparations confirms the absence of LPG in *Δlpg1*- derived SLAG and the presence of LPG in the SLAG of the WT and lpg1 add-back lines (**Figure S1**).

In addition, when platelets were incubated with SLAG obtained from WT, Δ*lpg1*-/+*LPG1* and Δ*lpg2*-/+*LPG*2 parasites in the presence of varying concentrations of PKC-alpha inhibitor, a significant reduction in secretion of DKK1 was observed at higher dose concentrations of PKC-alpha inhibitor. In contrast, DKK1 production from *Δlpg1*- and *Δlpg2*- SLAG-stimulated platelets exhibited some degree of inhibition by the PKC-alpha inhibitor at the higher concentrations (**Figures 6D, E**), but this was confirmed to be background platelet DKK1 that is being inhibited, as the non-stimulated platelets show a comparable level of inhibition (**Figure S2**). This suggests that the recognition of leishmania-derived LPG and induction of DKK1 from activated platelets may occur via the TLR1/2-MyD88 pathway.

### The lack of LPA formation induced by *Δlpg1*- and *Δlpg2*- parasites is restored in addback parasite (Δ*lpg1*-/+*LPG1* and Δ*lpg2*-/+*LPG*2) infected mice

Platelet-leukocyte interactions are indispensable events in hemostasis and inflammation (41, 42). Under inflammatory conditions, platelets interact with leukocytes, thus promoting their recruitment by the formation of platelet leukocyte aggregates via the interaction of PSGL-1 and P-selectin (28, 43). The consequence of this interaction enables leukocytes to fulfill their multiple cell-intrinsic functions and immunological tasks. We showed previously that pre-treatment of mice with DKK1 inhibitor 24 hours prior to infection with *L. major* reduced the elevation of LPA formation at 4 h post-infection (28). Furthermore, repeated administration of DKK1 inhibitor led to significant reduction in cellular recruitment at the site of infection and to the draining lymph node. These findings suggest that leukocyte platelet aggregation and infiltration of leukocytes to the infection site is driven by DKK1 production.

**Figure 7:**
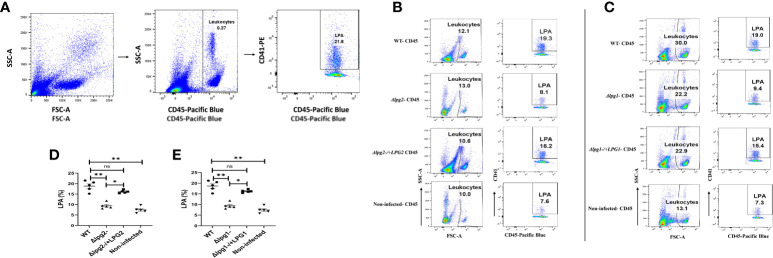
*Δlpg1*- and *Δlpg2*- parasites are ineffectual in inducing LPA formation. BALB/c mice were challenged with infective metacyclic promastigote (2 x 10^6^ parasites, n = 5) of WT, *Δlpg1*-, Δ*lpg2*-, Δ*lpg1*-/+*LPG1* and Δ*lpg2*-/+*LPG2* strains via the footpad. Control mice (n = 5) were given 0.9% NaCl saline. Blood was collected via retro-orbital sinus at day 3 PI. Blood samples were analyzed by flow cytometry for LPA. Representative flow cytometry dot plots showing the analyses of LPA performed on day 3 PI **(A)**. Representative dot plots shown in **(B, C)** are from concatenated samples of each experimental group, and the corresponding graphs **(D, E)** indicate the percentage of LPA molecules by CD45+ cells. A dot plot of each sample in all the experimental groups is presented in **Figure S4** **(A, B)**. In all the experiments, WT-infected and non-infected mice served as a positive and negative control, respectively. Results are presented as mean (± SEM). One-way ANOVA with Bonferroni’s *post hoc* test was performed to analyze the data *p < 0.05, **p < 0.01, ‘ns’ indicates not significant (p > 0.05).

Given that DKK1 production in mice infected with *Δlpg1*- and *Δlpg2*- parasites was impaired, we considered the possibility that the loss of *LPG1* and *LPG2* gene-dependent molecules might decrease LPA formation in blood obtained from *Δlpg1*- and *Δlpg2*- mutant infected mice (**Figures 7**). Relative to the WT-infected mice, LPA circulating in the blood was comparable to those observed in Δ*lpg1*-/+*LPG1* and Δ*lpg2*-/+*LPG2* infected mice, but this aggregate was significantly impaired in *Δlpg1*- and *Δlpg2*- infected groups at day 3 PI. In addition, the LPA level formed in response to infection with *Δlpg1*- parasites is comparable to those formed in response to infection with *Δlpg2*- parasites (**Figures 7A–E**). This suggests that LPG-activated platelets enhance P-selectin expression, resulting in platelet leukocyte aggregation.

## Discussion

The interactions between *Leishmania* and host cells have fundamental effects on the disease outcome (44). Leishmaniasis is thought to be initiated by direct parasitization of neutrophils and monocytic cells following parasite deposition into the skin (45–47). However, it has been demonstrated that platelets can be stimultated by *Leishmania* through activation of complement to produce PDGF, which is a potent inducer of CCL2 (MCP­1). MCP-1 promotes the recruitment of a subpopulation of effector monocytes to the infection site. Complement was found to be essential for the generation of platelet PDGF, which was found for the first 30-60 minutes at the site of *L. major* infection (48). Complement activation is also known to generate chemotactic peptides (C5a, C3a), recruiting polymorphonuclear cells (PMN), which are critical for the establishment of infection. Further, C5a has been shown recently to be important for PMN trafficking to the draining lymph node (49). We have previously shown that DKK1 released from activated platelets in response to *L. major* infection promoted a Th2 inflammatory response and leukocyte-platelet aggregation (28); this is important biologically for the recruitment or migration of PMNs across the vasculature (36, 50, 51). Further, inhibition of DKK1 *in vivo* or platelet depletion resulted in reduction in IL-4, IL-10 and *L. major* parasite burden as well as cellular recruitment to the draining lymph node and site of infection. These studies suggest that platelets are likely one of the first cells to initiate innate immune responses to *Leishmania*, and are important in determining the outcome of infection (48, 52, 53).

Therefore, the primary aim of this study was to determine the *Leishmania* surface molecules involved in platelet activation and in the subsequent DKK1 production from activated platelets that could help account for the pathological type 2 inflammation observed in mice infected with *L. major* (28). We focused initially on the glycoconjugates LPG and PPG and compared the contribution of *L. major Δlpg1*- and *Δlpg2*- parasites on platelet activation and subsequent DKK1 production in infected mice. Data show that the absence of PPG and LPG in the *Δlpg2*- mutant and deletion of LPG alone in the *Δlpg1*- mutant significantly influence the activation of platelets by impairing the expression of P-selectin and DKK1 production compared to the responses obtained from WT and add-back *L. major* infected mice. In addition, the percentage of LPA at day 3 PI in both *Δlpg1*- and *Δlpg2*- mutant infected mice was significantly lower than those obtained from WT controls. In contrast, mice infected with *Δlpg1*- and *Δlpg2*- parasites complemented with the *LPG1* and *LPG2* genes (Δ*lpg1*-/+*LPG1* and Δ*lpg2*-/+*LPG2*) manifested a phenotype similar to those infected with WT parasites. As *Δlpg1*- and *Δlpg2*- parasite infections result in relatively similar poor pathogenicity, implying that the deletion of PPG glycoconjugates did not influence the activation of platelets, instead LPG is the key molecule responsible for platelet activation.

*Leishmania* LPG has been reported to induce inflammation through TLR2 and 4 in macrophages and other cells (54–58). Notably, although there is considerable structural variation in leishmanial LPG between species (59), activation of TLR2 by LPG appears to be found across the genus (*L. (V.). braziliensis*, *L. mexicana*, *L.infantum*, *L. major*) (24, 57, 60–62). Although TLR4 activation does not lead to DKK1 release from platelets,the finding that platelet TLR2 mediates the induction of DKK1 suggests that this mechanism may play a role in the initiation of infection and pathogenesis of other *Leishmania* species (63–65).

However, previous studies have demonstrated that the LPG virulence factor is expressed and promotes the establishment of infection by metacyclic promastigotes in host cells; LPG expression significantly diminishes in amastigote stage (66–69). This suggests, as level of LPG expression declines in the mammalian host, it would have little/no critical function as the disease progresses. Since the expression of LPG decreases in the amastigote phase, it is unclear how P-selectin and DKKI observed in the WT and add-back infected mice on days 14 and 42 PI remained elevated. This may be associated with multiple factors. One possibility is the presence of residual membrane-anchored LPG, which might prolong platelet activation and DKK1 production. Previous studies showed that the down-regulation of the promastigote-specific virulence factor LPG varies in different host cell environments (68). Thus, the elevated DKK1 and P-selectin expression observed on days 14 and 42 PI could also be associated with prolonged retention of LPG virulence factor in platelets, or macrophages or possibly dendritic cells. Alternatively, studies demonstrate that TNF-alpha exerts its effects through TNF-RI expressed on megakaryocytes to activate platelets (70–74). Thus, the continuity of platelet activation and DKK1 production observed at later phase of infection may be associated with TNF-alpha activated platelets. Further, presence of *Leishmania* antigens and induction of immunoglobulins have been shown to induce the formation of circulating immune complexes (75). Antigen-antibody complexes are capable of activating platelets via interaction with FcγRIIa (76–78). Hence, as infection progresses the immune complexes formed could lead to sustained platelet activation and DKK1 release. Upon activation, platelets release certain complement components including C1q, C3, C4, and C5b-9 (79, 80), and they also express receptor for C3a, C5a, C1q and C4 (81–84). Previous studies reported that C3a/C5a and C1q binds to their respective ligands and enhance platelet activation (85, 86). This suggests that C3a and C1q may prolong platelet activation and DKK1 production. Inflammatory responses induced could also provide alternate modes for platelet activation. Collagen is a unique agonist and a potent activator for platelets (87). Glycoproteins Ia/IIa, glycoprotein VI and integrin α_2_β_1_, has been identified as the major platelet receptor for collagen (87–89). This indicates that collagen-activated platelets may as well provide a possible mechanism of sustained platelet activation. The WT, addback and mutant infected mice released DKK1 at day 42 PI, but the level of P-selectin expression was impaired in the mutant infected mice. Thus, DKK1 prodution and platelet activation dissociate at the later phase of infection. This suggests that by day 42 PI, the surviving amastigotes could be inducing the DKK1 via an alternate pathway (which may or may not be platelet-mediated). The mechanism of the sustained DKK1 release is not clear but of interest and requires further investigation.

*Leishmania* infection triggers cascades of events in macrophages that influence the ensuing immune response. One of the most crucial initial signaling events is interleukin-12 production by the infected macrophage, resulting in subsequent induction of Th1 response and production of interferon-gamma (IFN-γ) required to kill the pathogen (90–92). Unlike most microbial pathogens, *Leishmania* promastigotes have developed immune evasion strategies that prevent immediate “classical” macrophage proinflammatory activation (93). These strategies include engaging suppression-associated macrophage surface receptors such as complement receptor 3 (CR3) (94). Earlier studies of macrophages from CR3-deficient mice demonstrated that CR3 engagement is required for IL-12 suppression during *Leishmania* infection (95). In addition, *Leishmania* is known to delay antimicrobial activity in infected macrophages by inhibiting host defence mechanisms such as protein kinase C activation (96), and upregulation of inducible nitric oxide synthase following IFN-γ stimulation (92, 97). Thus, infected macrophages response to IFN-γ is repressed. Furthermore, it has been shown that *Leishmania donovani*-infected U937 cells exhibit decreased activation of the IFN-γ receptor (98, 99), and *L. donovani* amastigotes negatively influence the expression of major histocompatibility complex class II (MHC-II) following IFN-γ stimulation (99). Most importantly, *Leishmania* has been demonstrated to suppress TLR4-mediated proinflammatory cytokine production in BMDMs (100). Thus, these studies show that *Leishmania* evades the host immune response by inhibiting macrophage activation. Although *Leishmania* parasites interact and infect various host cell types- macrophages, neutrophils and dendritic cells are arguably the most important cells that regulate the outcome of infection (44, 101). However, there is limited information on the recruitment of these cells to the site of infection. Our study highlighted that DKK1 released by LPG-activated platelets regulates leukocyte platelet aggregation required for infiltration of leukocytes to the infection site. This is consistent with our earlier study that showed that inhibition of DKK1 diminished macrophage accumulation at the infection site, impaired Th2 polarization, and dampened parasitic load (28). This suggests that LPG-activated platelets might be the primary cells that significantly influence the initiation and outcome of infection.

In conclusion, our studies establish the importance of *L. major*-derived LPG in the induction of DKK1, which may serve as a novel immunomodulatory molecule regulating LPA formation (PMN recruitment) and chronic Th2 inflammatory response. This observation stresses the need to evaluate further the mechanism through which DKK1 facilitates leukocyte migration and LPA formation at the infection site.

## Data availability statement

The original contributions presented in the study are included in the article/**Supplementary Material**. Further inquiries can be directed to the corresponding author.

## Ethics statement

The animal study was approved by Arizona University Institutional Animal Care and Use Committee (IACUC). The study was conducted in accordance with the local legislation and institutional requirements.

## Author contributions

OI: Conceptualization, Data curation, Methodology, Validation, Writing – original draft, Writing – review & editing. AS: Validation, Writing – review & editing, Data curation, Formal Analysis, Methodology. SB: Validation, Writing – review & editing, Funding acquisition, Resources. DM-P: Conceptualization, Supervision, Validation, Writing – review & editing, Methodology. AB: Conceptualization, Funding acquisition, Resources, Supervision, Validation, Visualization, Writing – review & editing.
