# Supplementary material for: Leishmania major-derived lipophosphoglycan influences the host’s early immune response by inducing platelet activation and DKK1 production via TLR1/2
Source: Front Immunol. 2023 Oct 11;14:1257046. doi: 10.3389/fimmu.2023.1257046 (PMC10598878; doi:10.3389/fimmu.2023.1257046)
Supplement: Supplementary file 1 [file DataSheet_1.docx]

**Supplemental Material**

***Leishmania major-*derived lipophosphoglycan influences the host’s early immune response by inducing platelet activation and DKK1 production via TLR1/2**

**Olivia C. Ihedioha*, Anutr Sivakoses, Stephen M. Beverley, Diane McMahon-Pratt, Alfred L.M. Bothwell**

*** Correspondence:** Alfred L. M. Bothwell: abothwell@arizona.edu


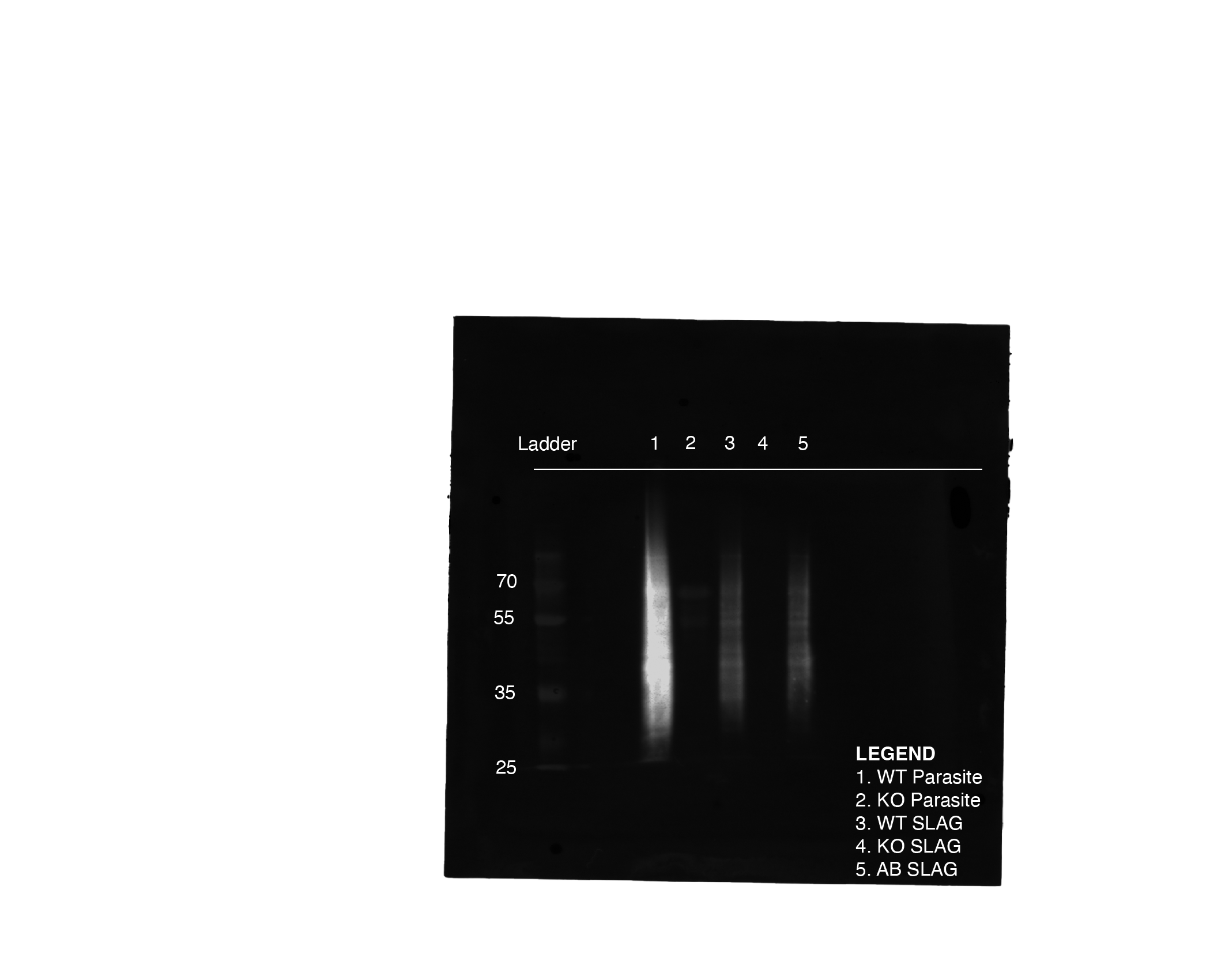


**Fig. S1**

**Figure S1: Expression of LPG is absent in *Δlpg1*- line.** Shown are the results of Western blot analyses of SLAG (7 µg) preparations from either WT, *Δlpg1* (KO)*^-^, or* Δ*lpg1*-/+*LPG1* (AB) parasites. 10% SDS-PAGE and Western blots were performed as indicated in the Methods Section. Following blocking of PVDF membrane with 5% Milk in TBS-Tween20 for 1 hour, blots were probed with WIC79.3 antibody (1:1000 diluted in 5% BSA-TBS-Tween20) and incubated overnight. After washing in TBST, the membrane was incubated for 1 h with anti-mouse IgG conjugated with HRP (1:1000 (Invitrogen)) and the reaction was visualized using Pico Substrate Kit (Thermofisher). The expression of LPG in SLAG from WT and Δ*lpg1*-/+*LPG1* parasites was absent in SLAG from *Δlpg1^-^* strains. (WT- Wild type; AB- add back, KO- knock out).



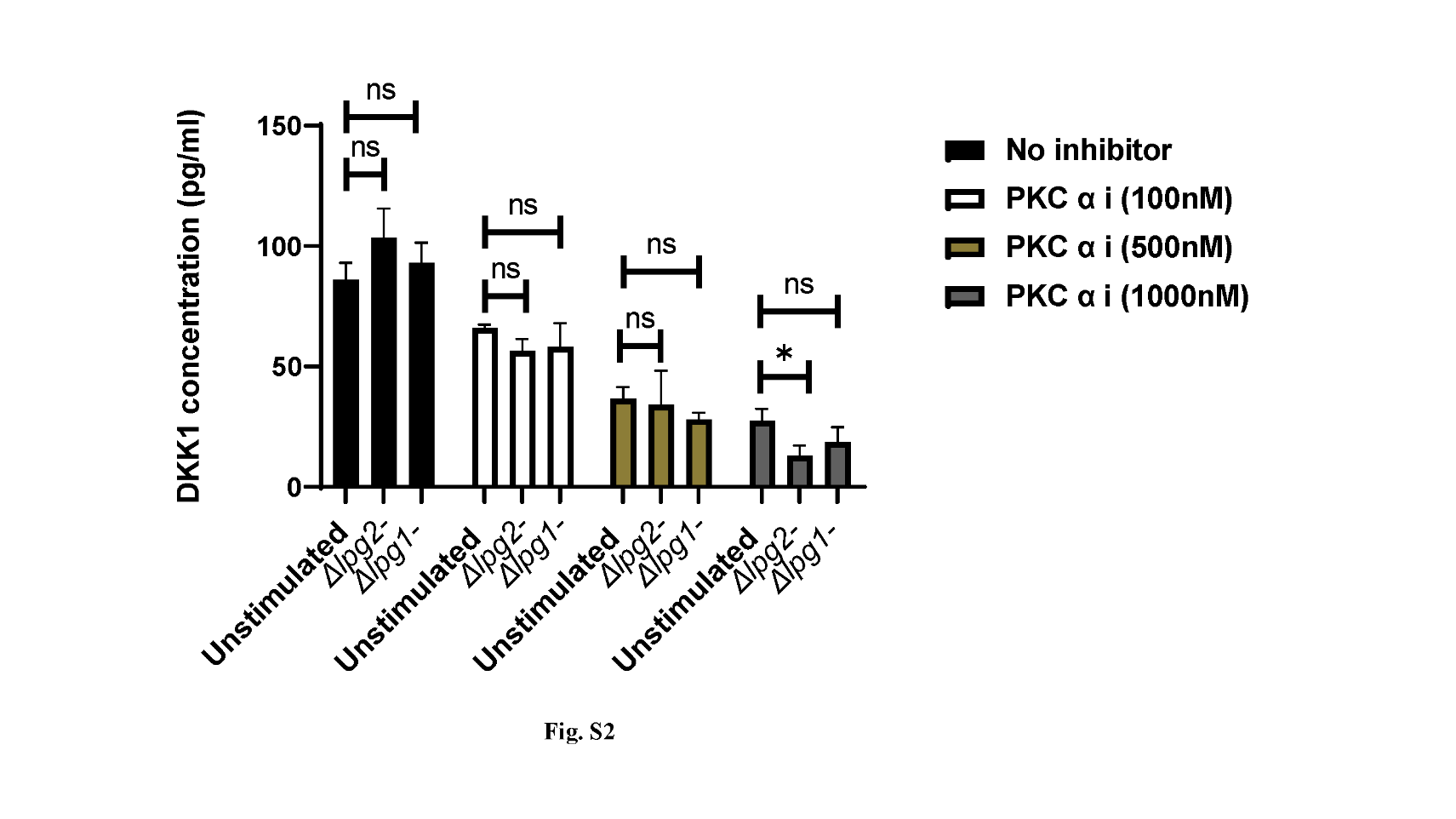


**Figure S2: Dose-dependent decrease in spontaneous/background DKK1 production in supernatant of unstimulated,** ***Δlpg1*- and *Δlpg2*- parasites activated platelets in the presence of various concentrations of PKC-alpha inhibitor.** Different concentrations of PKC-alpha inhibitor (Go 6976 PKC-alpha inhibitor (Abcam)) were used to treat platelets. Comparable levels of DKK1 release were found for Δ*lpg1*- and *Δlpg2*- parasite activated platelets and that found for background release throughout the dose range employed. DKK1 production was determined using ELISA.


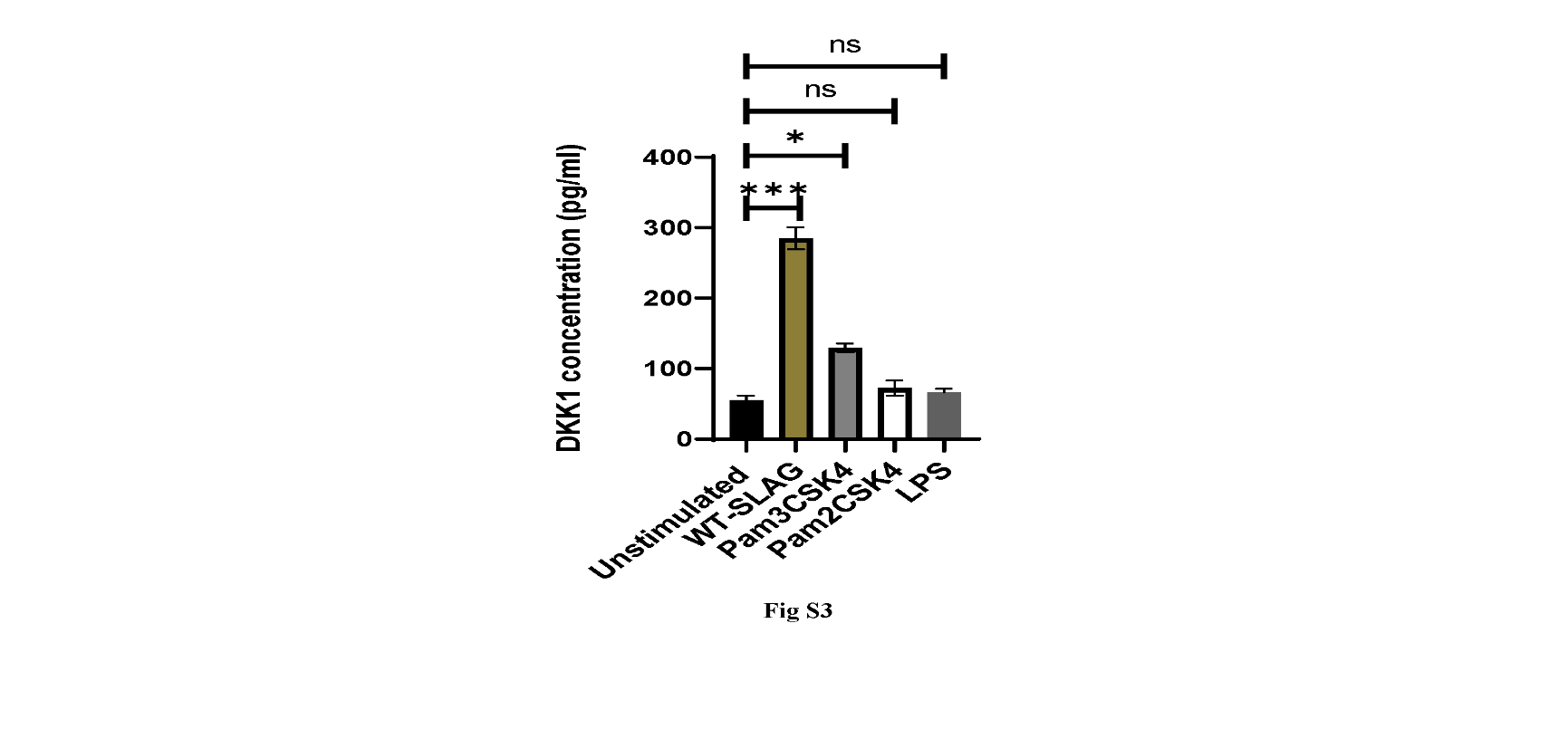


**Figure S3: LPS (TLR4) and Pam2CSK4 (TLR2/6) fail to induce DKK1.** SLAG and different TLR ligands (TLR1/2 (Pam3CysK4), TLR 2/6 (Pam2CysK4) and TLR4 (LPS) were used to stimulate naïve platelets and DKK1 production was determined using ELISA. Methods are as described in the Materials and Methods section. Only stimulation with SLAG and Pam3CSK4 induces the release of DKK1 from platelets. DKK1 was determined using ELISA.


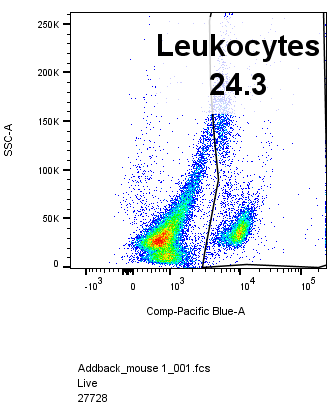

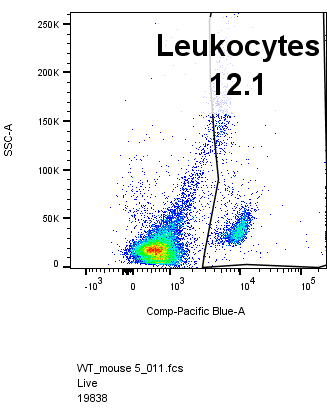

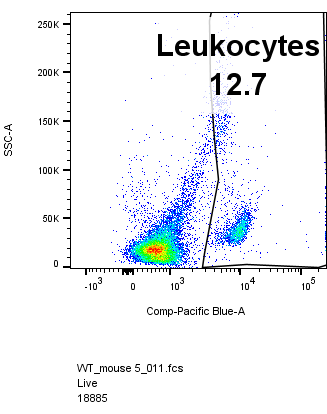

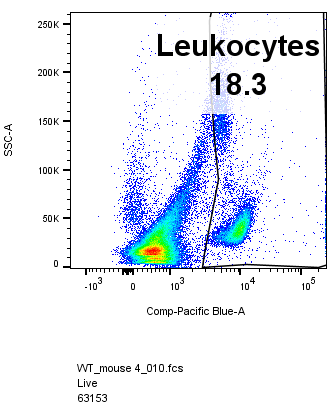

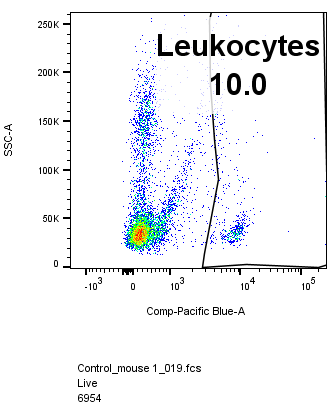

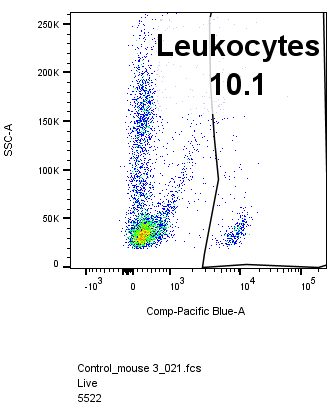

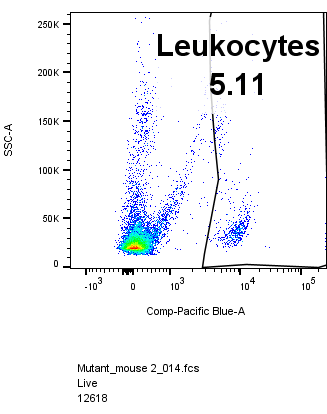

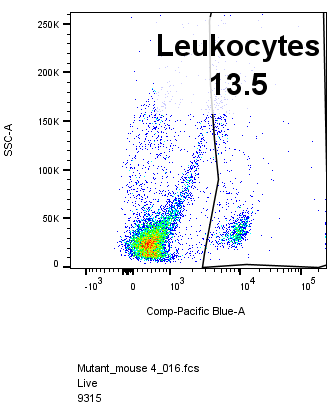

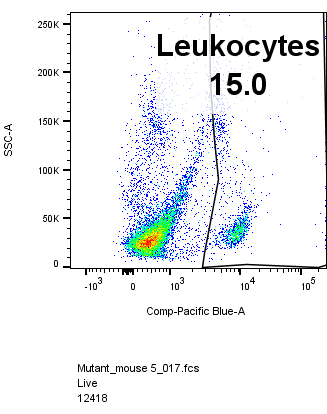

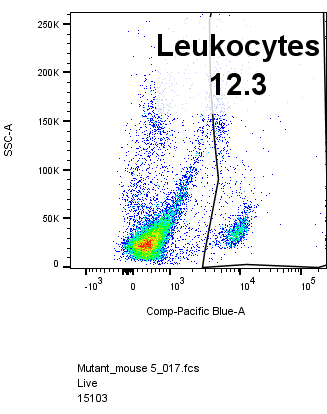

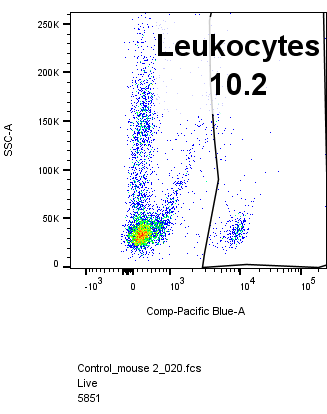

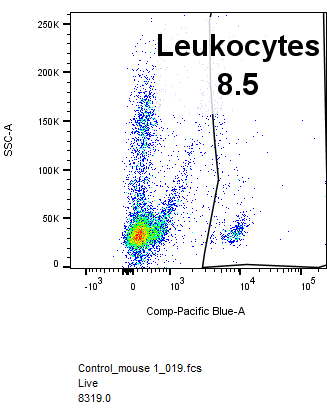

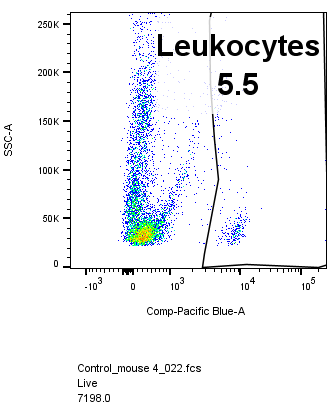

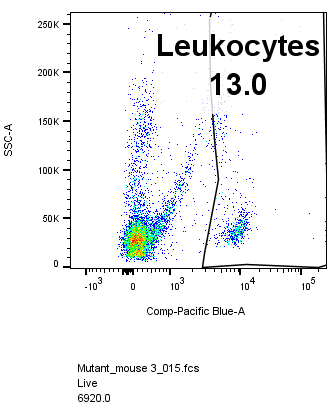

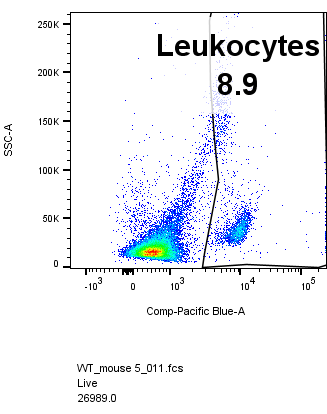

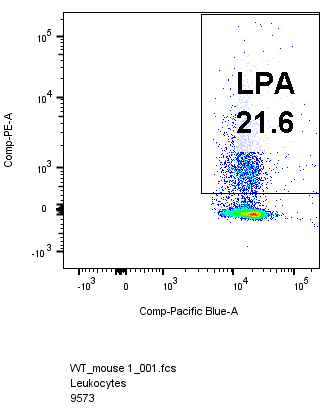

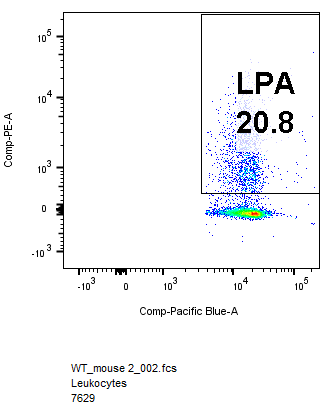

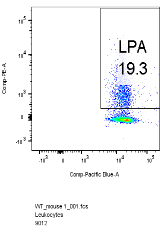

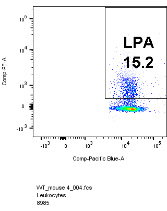

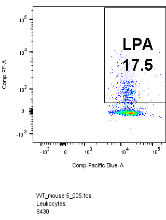

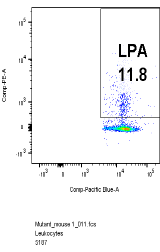

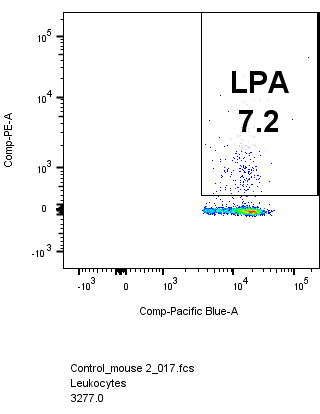

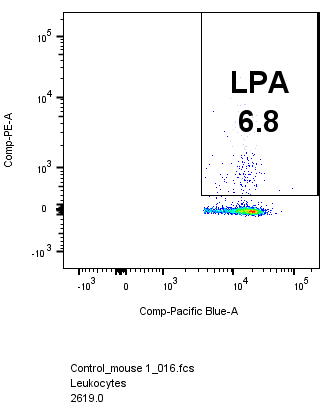

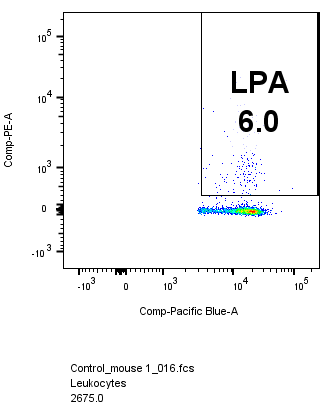

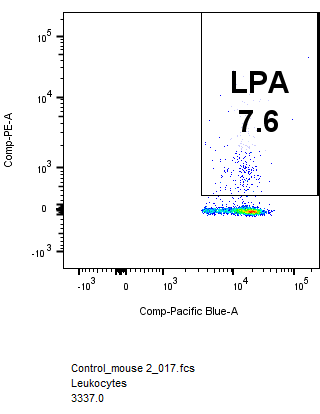

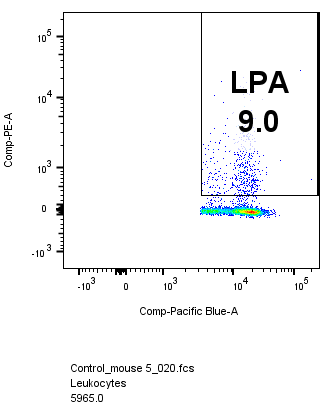

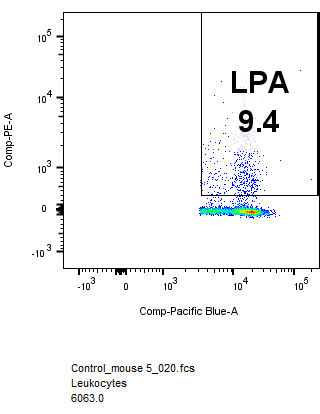

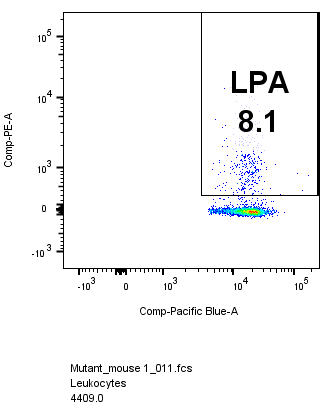

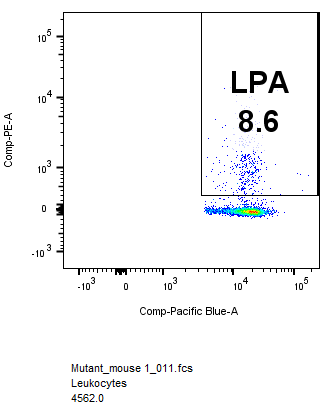

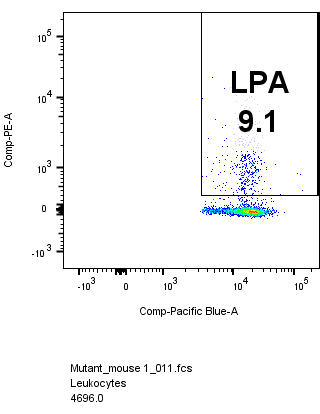


***Δlpg2-/+LPG2 CD45***

**Non-infected- CD45**

***Δlpg2- CD45***

**WT- CD45**

**CD41**


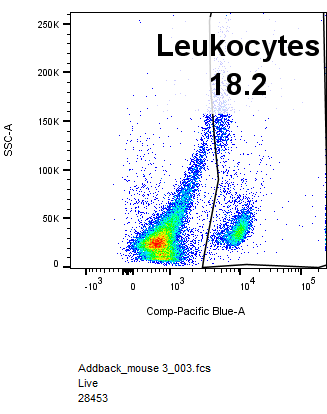

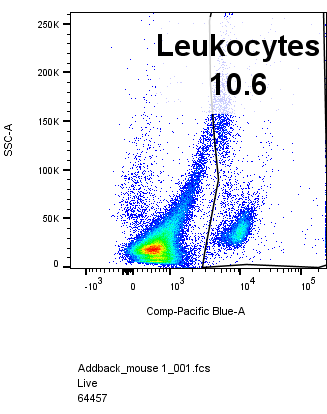

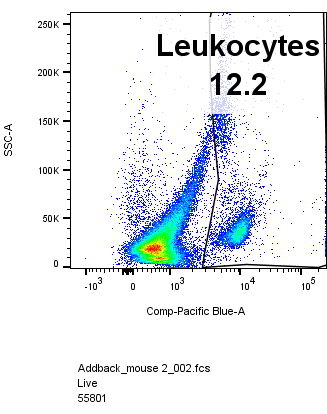

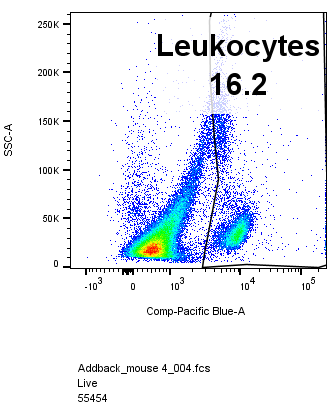

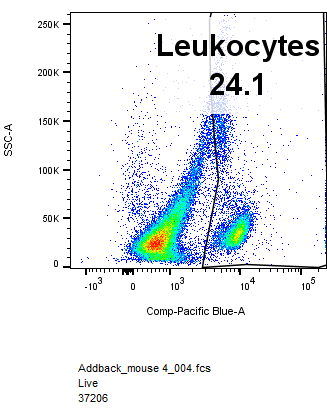

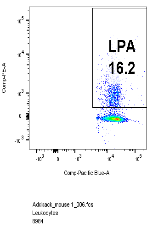

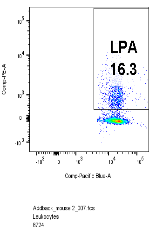

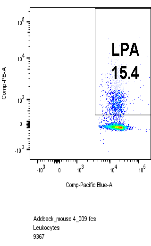

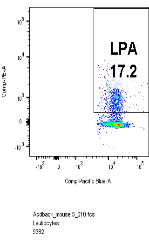

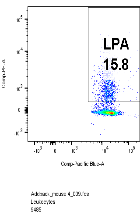


**CD41**

**CD41**

**CD41**

**SSC-A**

**SSC-A**

**SSC-A**

**SSC-A**

**A**


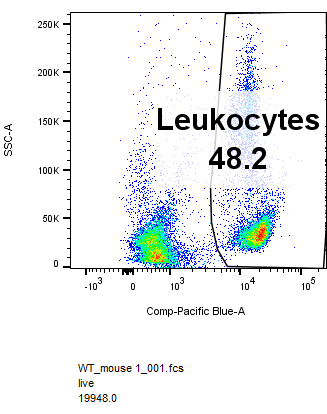

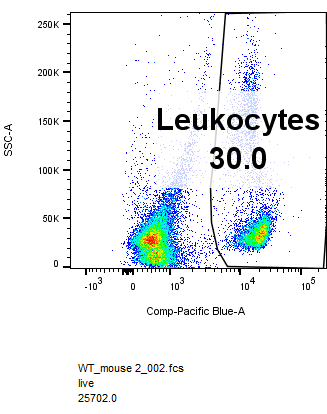

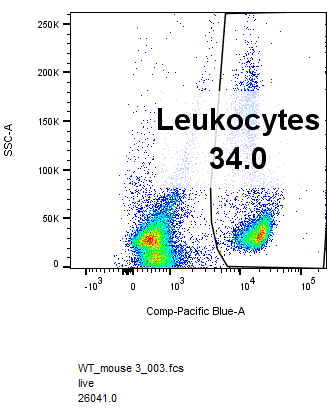

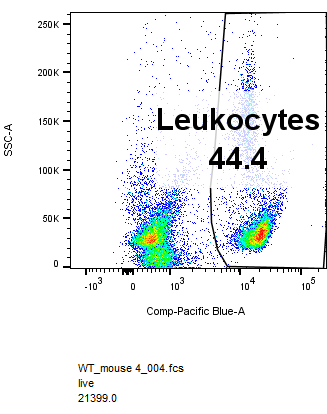

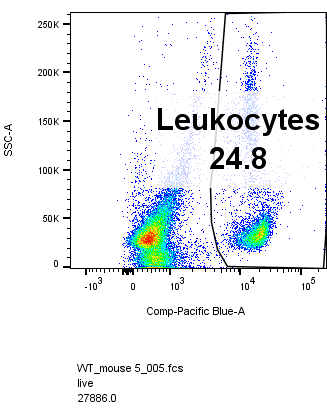

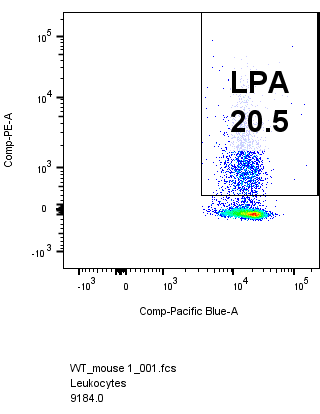

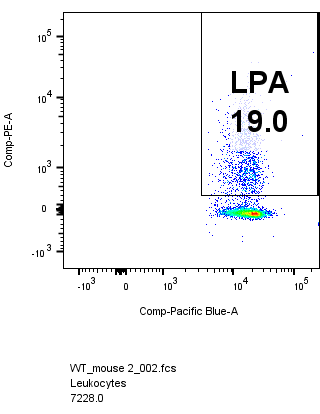

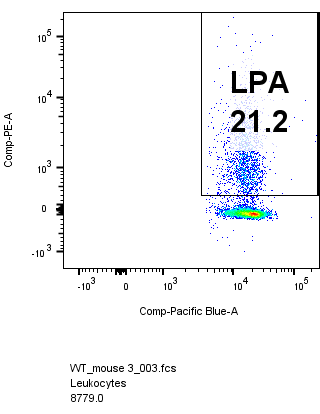

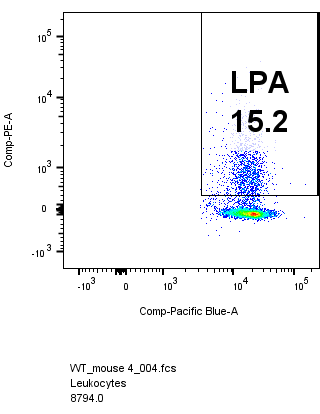

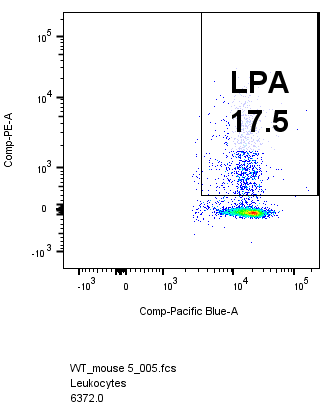

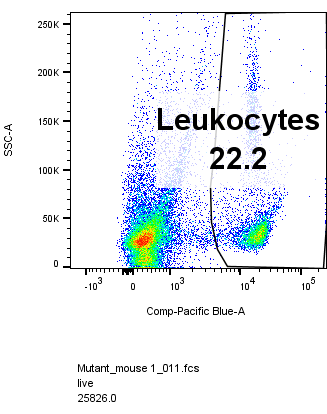

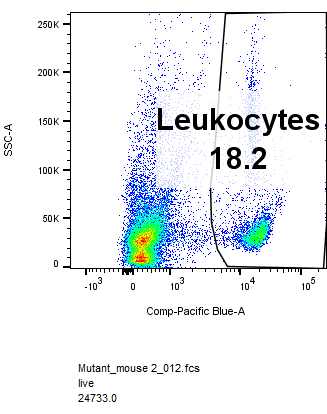

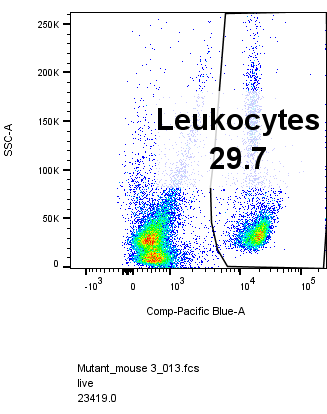

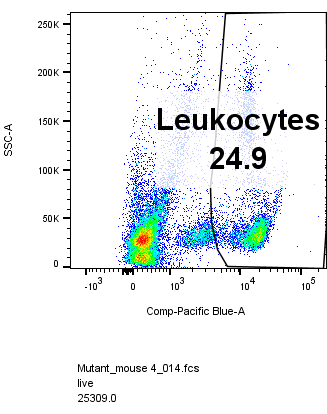

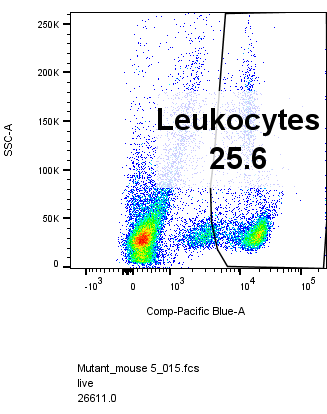

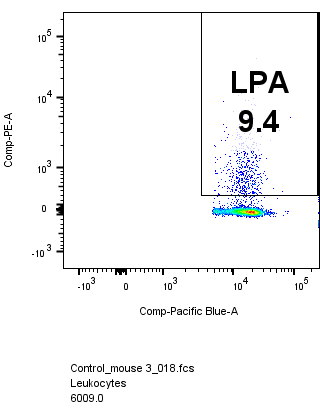

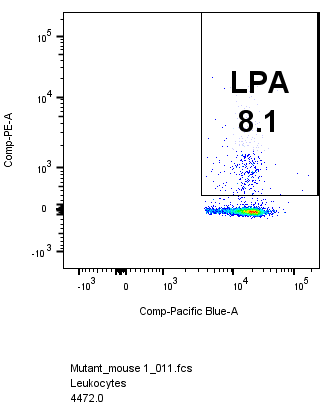

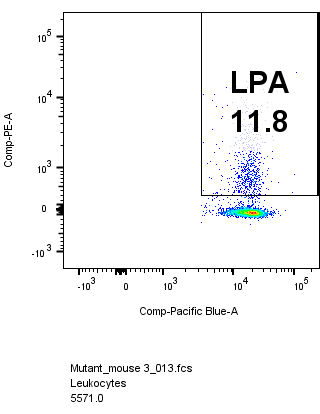

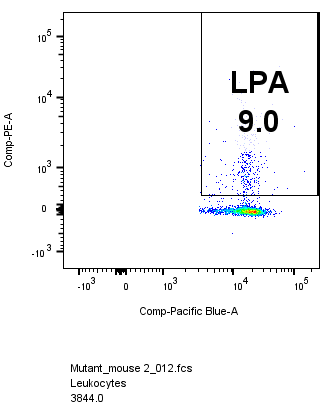

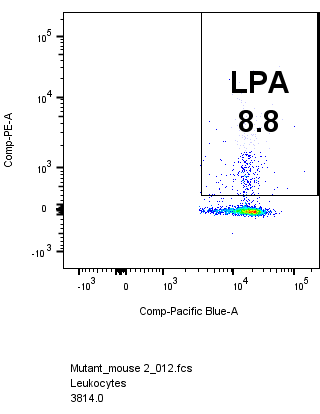


**CD41**


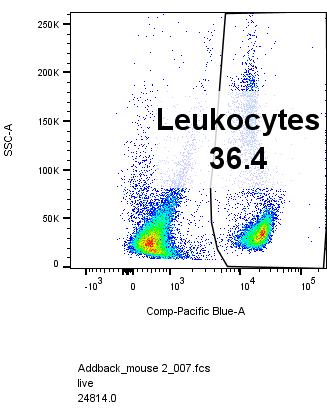

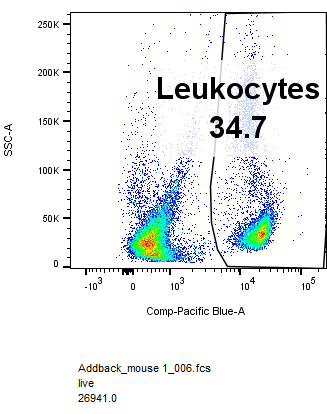

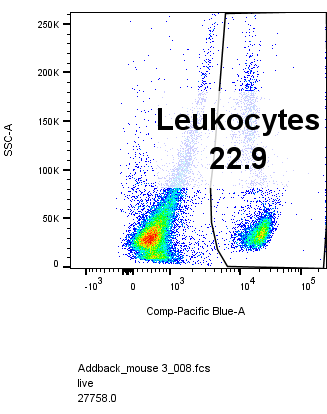

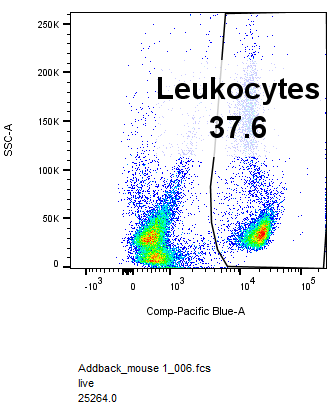

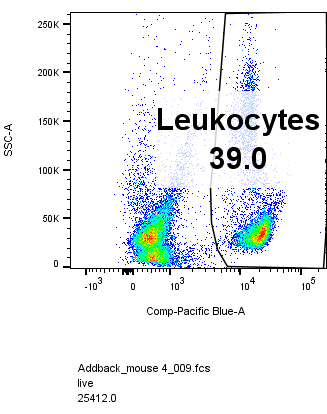

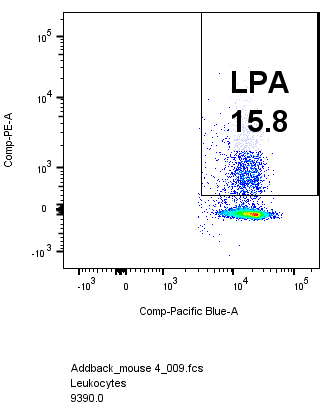

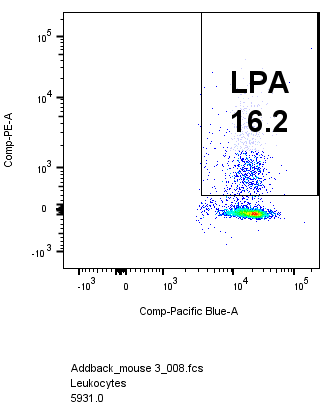

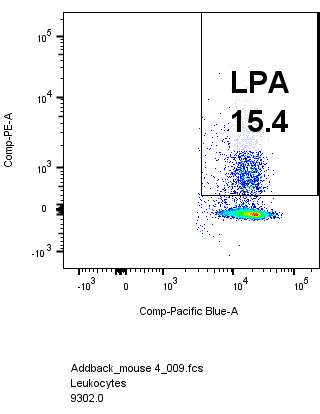

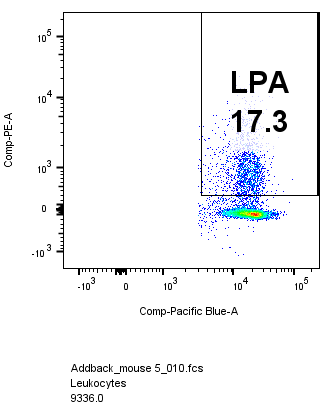

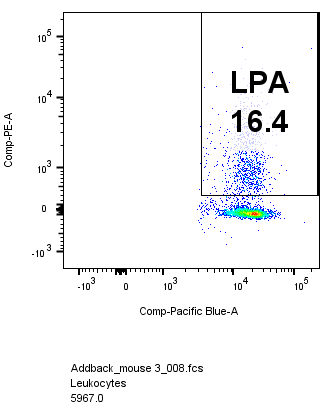


**CD41**


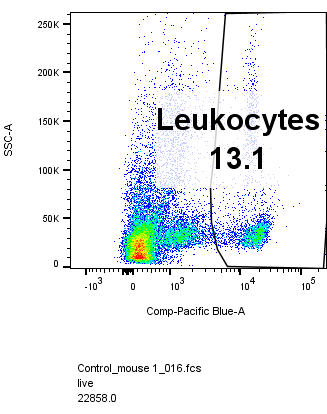

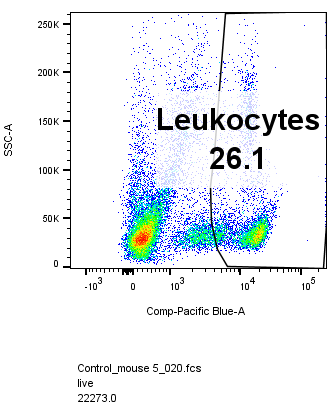

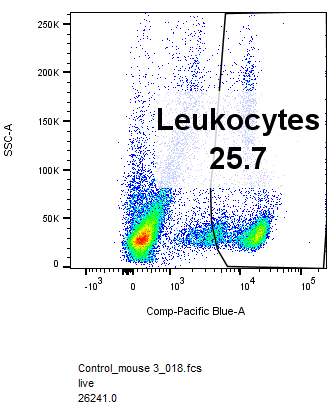

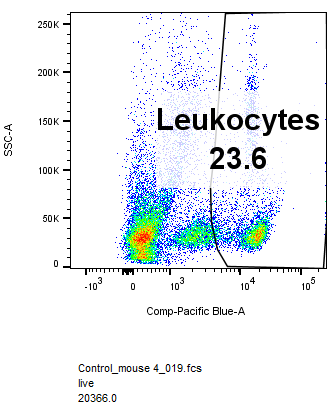

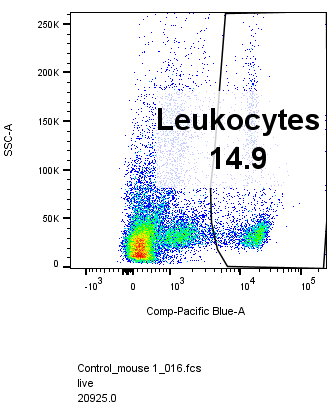

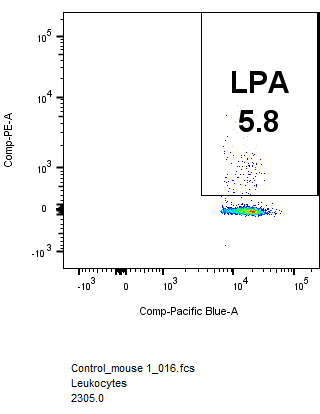

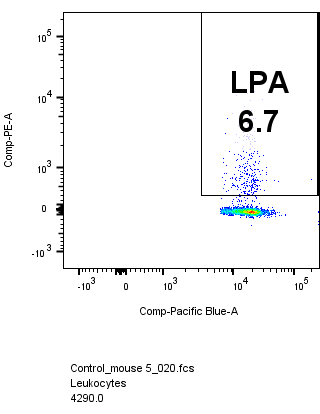

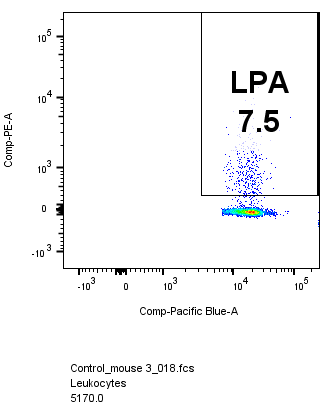

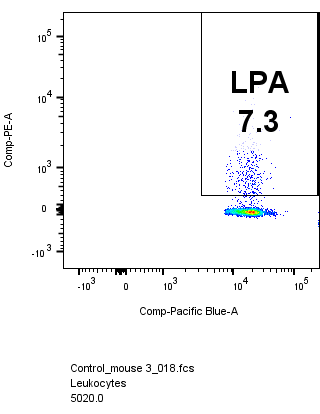

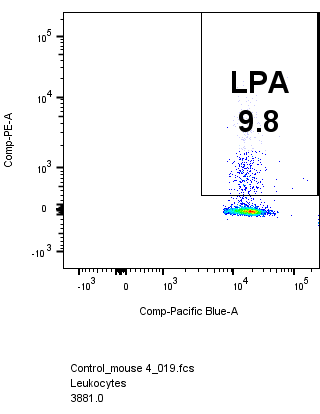


**CD41**

**CD41**

**WT- CD45**

***Δlpg1-/+LPG1- CD45***

**Non-infected- CD45**

***Δlpg1- CD45***

**SSC-A**

**SSC-A**

**SSC-A**

**SSC-A**

**B**

**Fig. S4:**  ***Δlpg1*- and *Δlpg2*-** **parasites are less effective in inducing LPA formation.**

BALB/c mice were challenged with infective metacyclic promastigote (2 x 10^6^ parasites, n = 5) of WT, *Δlpg1*-, Δ*lpg2*-, Δ*lpg1*-/+*LPG1* and Δ*lpg2*-/+*LPG2* strains via the footpad. Control mice (n = 5) were given 0.9% NaCl saline. Blood was collected via retro-orbital sinus at day 3 PI. Blood samples were analyzed by flow cytometry for LPA. Dot plots **(A) & (B)** indicate the percentage of LPA molecules by CD45+ cells in each of the samples from the different experimental groups. In all the experiments, WT-infected and non-infected mice served as a positive and negative control, respectively.
